# Improvements in Functioning with Esketamine Nasal Spray versus Quetiapine Extended Release in Patients with Treatment Resistant Depression

**DOI:** 10.1192/j.eurpsy.2025.1380

**Published:** 2025-08-26

**Authors:** E. Vieta, N. Ahmed, C. Arango, A. J. Cleare, K. Demyttenaere, M. Dold, T. Ito, Y. Kambarov, S. Krüger, P. M. Llorca, R. S. McIntyre, G. Sani, C. von Holt, B. Rive

**Affiliations:** 1 Institute of Neuroscience, University of Barcelona, Hospital Clinic, IDIBAPS, CIBERSAM, Barcelona, Spain; 2 Sakina Mental Health & Wellbeing Services; College of Medicine, and Health Sciences of the United Arab Emirates University, Khalifa University, Abu Dhabi, United Arab Emirates; 3 Department of Child and Adolescent Psychiatry, Institute of Psychiatry and Mental Health, Hospital General Universitario Gregorio Marañón, School of Medicine, Universidad Complutense de Madrid, Instituto de Investigación Sanitaria Gregorio Marañón (IiSGM), CIBERSAM, Madrid, Spain; 4 Institute of Psychiatry, Psychology & Neuroscience, King’s College London, London, United Kingdom; 5 Universitair Psychiatrisch Centrum KU Leuven, Leuven, Belgium; 6 Department of Psychiatry and Psychotherapy, Medical University of Vienna, Vienna, Austria; 7 Janssen EMEA, High Wycombe, United Kingdom; 8 Janssen EMEA, Beerse, Belgium; 9 Vivantes Humboldt Clinic, Department of Mental Health, Berlin, Germany; 10 CHU Clermont-Ferrand, Department of Psychiatry, University of Clermont Auvergne, Clermont-Ferrand, France; 11 University of Toronto; 12 Braxia Scientific, Toronto, Canada; 13 Department of Neuroscience, Section of Psychiatry, Università Cattolica del Sacro Cuore; 14 Department of Psychiatry, Fondazione Policlinico Universitario Agostino Gemelli IRCCS, Rome, Italy; 15 Janssen EMEA, Neuss, Germany; 16 Janssen EMEA, Paris, France

## Abstract

**Introduction:**

In the ESCAPE-TRD study, esketamine nasal spray (ESK-NS) significantly increased chance of remission at Week 8 versus (vs) quetiapine extended release (Q-XR) in patients (pts) with treatment resistant depression (TRD; Reif *et al*. NEJM 2023; 389:1298–309). Changes in disability and functional impairment due to depressive symptoms assessed with the Sheehan Disability Scale (SDS) are reported.

**Objectives:**

To assess the effect of ESK-NS vs Q-XR on pts’ daily functioning using SDS, considering their symptom evolution.

**Methods:**

ESCAPE‑TRD was a randomised phase IIIb trial comparing the efficacy of ESK-NS vs Q-XR, both alongside an ongoing selective serotonin/serotonin-norepinephrine reuptake inhibitor, in pts with TRD. Clinical response (CRes) was defined as ≥50% improvement in Montgomery-Åsberg Depression Rating Scale (MADRS) score from baseline or total score ≤10, clinical remission (CRem) was defined as total MADRS score of ≤10, and functional remission (FRem) was defined as SDS total score ≤6. The Kaplan-Meier method was used for time to event analyses, and hazard ratios (HRs) were estimated using Cox regression models. Time in each state was estimated by treatment arm and compared between arms using analysis of covariance.

**Results:**

336 and 340 pts were randomised to ESK-NS and Q-XR, respectively. Significantly more ESK-NS treated pts achieved CRes, CRem and FRem (HRs: 1.848, 1.711 and 1.819, respectively; all p<0.001), and achieved these faster, compared to Q-XR (**Figure 1**). In each arm and at each time point, more pts reached CRes than CRem, and more reached CRem than FRem, illustrating that FRem is more difficult to achieve (**Figure 1**). Total time in CRes was 5.4 weeks greater for ESK-NS compared with Q-XR; total time in CRem was 3.7 weeks greater and in FRem 2.0 weeks greater for ESK-NS vs Q-XR, respectively (**Table 1**).

**Image 1:**

**Figure:**
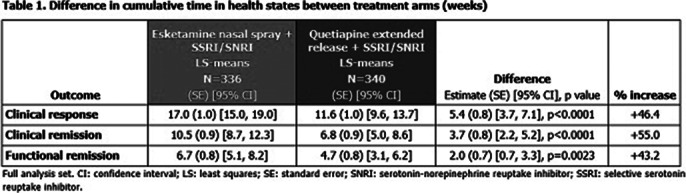

**Image 2:**

**Figure:**
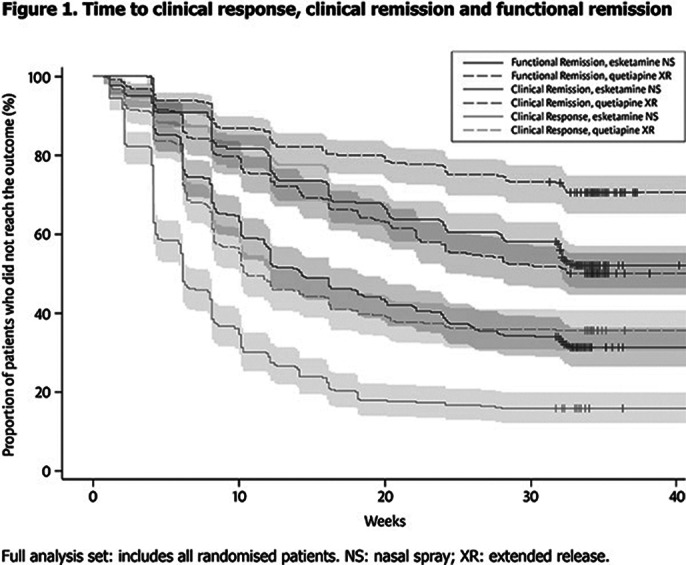

**Conclusions:**

These data support a temporal cascade of events from CRes to CRem to FRem; ESK-NS improved time to, and in, each outcome vs Q-XR. Treatments that reduce clinical symptoms better and faster provide the best chance of improving functional impairment.

**Disclosure of Interest:**

None Declared

